# Study of the Pollen Grain Metabolome under Deposition of Nitrogen and Phosphorus in *Taxus baccata* L. and *Juniperus communis* L.

**DOI:** 10.3390/ijms232214105

**Published:** 2022-11-15

**Authors:** Emilia Pers-Kamczyc, Jacek Kamczyc

**Affiliations:** 1 Department of Genetics and Environmental Interactions, Institute of Dendrology, Polish Academy of Sciences, 5 Parkowa Str., 62-035 Kórnik, Poland; 2 Department of Game Management and Forest Protection, Faculty of Forestry and Wood Technology, Poznan University of Life Sciences, 71 Wojska Polskiego Str., 60-625 Poznań, Poland

**Keywords:** quality of pollen grain, trees, pollen metabolome, nitrogen deposition, pollen germination, gymnosperm

## Abstract

Nitrogen plays an important role in both quantitative and qualitative aspects of plant reproduction, including pollen grain compounds and seed production. Recent studies have pointed out that pollen grains produced by male plants of *T. baccata* and *J. communis* subjected to a long period of fertilizer supplementation have lower in vitro germination ability and higher nitrogen content. To gain molecular insights into these observations, we conducted GC–MS analysis of both species to characterize the metabolomes of dry, mature pollen grains, which allowed for the identification and quantification of more than 200 metabolites. The results demonstrated that fertilizer supplementation impacts the relative content of 14 metabolites in *J. communis* (9 downregulated and 5 upregulated) and 21 in *T. baccata* (6 downregulated and 15 upregulated). Although plants showed little similarity in patterns, in metabolite profiles, both up and down fold-changes were observed. This is the first report on the gymnosperm pollen grain metabolomic profile and changes induced by long-term nitrogen and phosphorus supplementation. Pollen grains produced by fertilizer-supplemented male individuals had significantly lower relative content of linolenic acid, 5,6-dihydrouracil, maltotriose, galactonic acid, D-xylulose, and glycerol-α-phosphate but higher content of sorbitol, glucosamine, and 1,5-anhydro-D-glucitol as well as n-acetyl-d-hexosamine, dimethyl phthalate, glycine, galactose-6-phosphate, D-fructose-6-phosphate, pyroglutamic acid, and 3-(3-hydroxyphenyl)-3-hydroxypropionic acid. Thus, in pollen grain samples earlier shown to have different germination abilities, the presence of different metabolites indicates a significant environmental impact on the quality of gymnosperm pollen grains.

## 1. Introduction

Nitrogen (N) availability in the forest is considered the primary growth-limiting factor in most of the European continental and northern forests [1,2], although other nutrients, such as phosphorus (P) [3] and potassium (K) [4], are also very important for plant growth and development. Although fertilizer supplementation is not widely used in forestry, interest has been increasing due to its application in timber production [5]. Moreover, recent studies have pointed to the increasing deposition of anthropogenic N and P, and N deposition is projected to significantly increase in the next 30 years compared with the 1900s [6], and nitrogen deposition in soil is also found to be currently increasing [7]. N deposition has been indicated as a key driver for increased forest growth, in addition to widespread tree mortality and decreased plant biodiversity [8]. The reason for the higher nutrient deposition is related to the excessive and unwarranted use of fertilizers, and many areas worldwide have been affected by excessive nitrogen levels [9].

In our previous studies, we showed that an increase in nutrient availability improves the reproductive potential of female *Taxus baccata* L. and *Juniperus communis* L. plants by increasing the number of produced seeds and seed mass [10]. Moreover, increased male reproductive potential was also observed, and thus the productivity of male flowers and produced pollen grains when plants were grown in soil conditions of higher nutrient availability (especially nitrogen, phosphorus, potassium, and magnesium) was at least two times higher compared with their non-fertilized counterparts [11,12]. However, there were also negative consequences; namely, plants receiving additional fertilizer for a longer time were characterized by significantly lower quality of produced pollen grains and seeds. The in vitro germination rates of pollen grains are significantly lower in fertilized plants than non-fertilized plants. Similarly, long-term fertilization improves the productivity of seeds by female *T. baccata* plants, but they are characterized by lower germination ability [10,13]. Nutritional availability was shown to affect the chemical composition of both pollen grains and seeds and the metabolome profiles of fertilized seeds compared with their non-fertilized counterparts [10,11,12,13].

Untargeted metabolomics is a powerful tool for detecting numerous metabolites within a tissue and allows comparison of the metabolite profiles between analyzed samples. Thus, environmental as well as genetic effects and their interactions on pollen grains’ development can also be investigated [14,15]. Metabolomics helps to identify and quantify the complete set of metabolites present in a cell at a specific time and has been used to determine the potential allergen content in pollen grains [16]. Metabolite profiling with untargeted gas (or liquid) chromatography coupled with mass spectrometry can be used to detect and quantify numerous metabolites (e.g., sugars, amino acids, organic acids, and secondary metabolites). It allows for comprehensive qualitative and quantitative analysis, which further helps in understanding plant physiology and development or stress reactions [17].

Pollen grains are the sexual reproductive units of plants; therefore, their quantity plays an important role in seed production and population stability. The effect of pollen quality and quantity can have a wide-ranging impact on the reproduction of wind-pollinated trees, as pollen grains can migrate for very long distances [18,19,20]. Therefore, changes in pollen grain productivity and quality can have an ecological and evolutionary impact on the future of plant populations [21,22,23]. Pollen quality can be described in terms of volume or shape, pollen tube growth, enzymatic activity, and the carbon-to-nitrogen ratio [12,24,25,26]. Pollen grains have also recently been analyzed using metabolomic approaches to describe the developmental changes occurring within [14,27,28].

In the present study, we continue our research on the long-term effects related to nutrient availability on the male reproductive features of *T. baccata* and *J. communis* plants. Both species can be described as evergreen, wind-pollinated, dioecious gymnosperm plants but have different life histories [29,30]. We have chosen evergreen trees as a model species as evergreen species do not shed needles each year and therefore elements are accumulated in needles for a longer period; furthermore, a visible environmental impact on plant growth may be observed. Therefore, because we observed a negative impact of higher nutritional availability of nitrogen, phosphorus, and other elements on the germination ability of coniferous, wind-pollinated trees, we decided to analyze the metabolome profiles of dry, mature pollen grains that differed in their in vitro germination ability. We assumed that the higher content of nitrogen in the pollen grains of long-term-fertilized *T. baccata* and *J. communis* plants would result in differences compared with their non-fertilized counterparts and that the two species would have a similar pattern of differentiating metabolites when comparing corresponding treatments.

## 2. Results

### 2.1. Pollen Grain Metabolites

**Figure 1:**
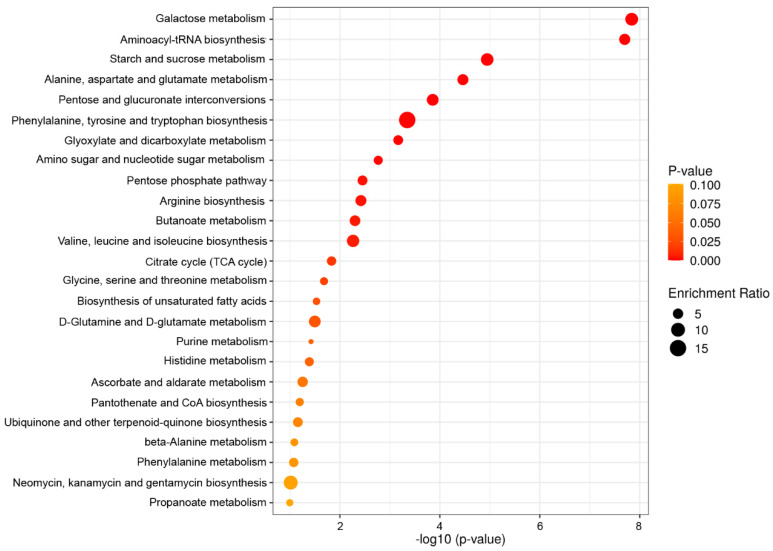
Results of over-representation analysis of the metabolite compounds present in *Juniperus communis* L. and *Taxus baccata* L. pollen grains. The red color indicates a significant level (*p* < 0.01) while the yellow color indicates a significant level at *p* = 0.10. The circles represent the number of metabolites involved or enriched in the pathway. The enrichment ratio refers to the ratio of the number of metabolites detected by GC–MS analysis to the total number of metabolites annotated by the pathway. The enrichment overview presents the top 25 metabolic pathways.

**Figure 2:**
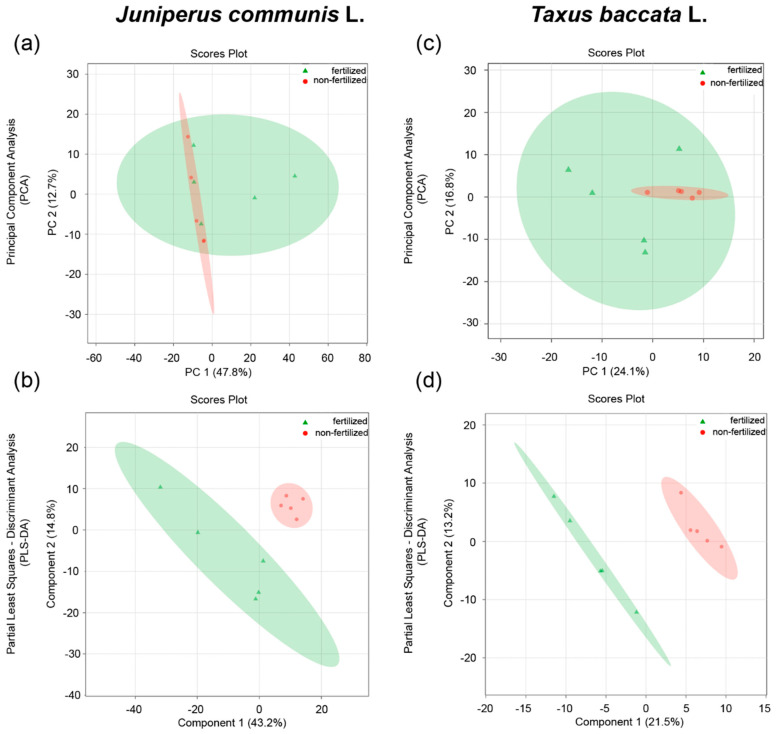
Results of the metabolome analysis of (**a**,**b**) *Juniperus communis* L. and (**c**,**d**) *Taxus baccata* L. pollen grains. (**a**,**c**) Principal component analysis scores—plot between the selected principal components (PC). (**b**,**d**) Partial least squares discriminant analysis (PLS-DA)—plot between the selected principal components (PC) showing separation between pollen grains collected from plants grown in fertilized (green) and non-fertilized (red) conditions. The percentages of explained variance are shown in brackets.

GC–MS analysis indicated the presence of 224 metabolites in the pollen grains of both species (Table S1). Metabolites from pollen grains were engaged in 56 metabolomic pathways (Table S2). The numerous identified metabolites are predicted to be involved in galactose metabolism (sucrose, raffinose, melibiose, α-lactose, glucose 1-phosphate, D-fructose, galactitol, glycerol, sorbitol, and myo-inositol), aminoacyl-tRNA biosynthesis (L-aspartate, L-phenylalanine, glycine, L-aspartic acid, L-serine, L-valine, L-alanine, L-lysine, L-isoleucine, L-threonine, L-tryptophan, L-tyrosine, L-proline, and L-glutamic acid), starch and sucrose metabolism (D-fructose, sucrose, glucose, 1-phosphate, glucose 6-phosphate, trehalose, D-maltose, and fructose 6-phosphate), alanine, aspartate, and glutamate metabolism (L-aspartic acid, L-asparagine, L-alanine, L-glutamic acid, and γ-aminobutyric acid (GABA), citric acid, succinic acid, and oxoglutaric acid), glyoxylate and dicarboxylate metabolism (glycolic acid, citric acid, L-serine, glycine, L-glutamic acid, and isocitric acid), amino sugar and nucleotide sugar metabolism (N-acetyl-D-glucosamine, N-acetylmannosamine, glucose 1-phosphate, fructose 6-phosphate, glucose 6-phosphate, D-fructose, and glucosamine), phenylalanine, tyrosine, and tryptophan biosynthesis (L-phenylalanine, L-tyrosine, and 4-hydroxyphenylpyruvic acid), pentose and glucuronate interconversions (D-xylulose, L-arabinose, D-xylitol, glucose 1-phosphate, D-xylose, and D-glucuronic acid), the pentose phosphate pathway (glucose 6-phosphate, D-ribose 5-phosphate, gluconic acid, and glyceric acid), arginine biosynthesis (L-glutamic acid, L-aspartic acid, oxyglutaric acid, and urea), and butanoate metabolism (GABA, L-glutamic acid, oxyglutaric acid, and succinic acid) (Figure 1 and Figure 2).

### 2.2. Effect of Long-Term Fertilization on Metabolites of Pollen Grains

In all samples of both analyzed species, all 224 metabolites were detected using a nontargeted GC–MS metabolomic strategy. In both analyzed species, partial least squares discriminant analysis (PLS-DA), but not unsupervised PCA, showed separations between treatment groups (Figure 2). Long-term treatment of paternal plants with nitrogen, phosphorus, and other minerals influenced the metabolites of *J. communis* and *T. baccata* pollen grains, as demonstrated by the clear separation between pollen grains collected from fertilized and non-fertilized plants (Figure 2b,d).

In *J. communis*, unpaired fold change (FC) analysis with an FC threshold equal to 2.0 pointed to the presence of metabolites with at least two-fold differences between fertilized and non-fertilized pollen grains. Pollen grains collected from fertilized *J. communis* plants were characterized by the presence of 106 downregulated and 47 upregulated metabolites compared with their non-fertilized counterparts (Table S3). However, *t*-test analysis with equal group variance of raw data identified 14 metabolites that were differentially expressed between experimental groups (*p* < 0.05), but none could be classified as differential when data were analyzed based on the false discovery rate (FDR). Combining the results of fold change analysis and t-tests in a volcano plot allowed the selection of nine downregulated and five upregulated metabolites in fertilized vs. non-fertilized pollen grains when raw data were used (Table 1 and Table S1), and none when based on FDR (*p* < 0.05).

**Figure 3:**
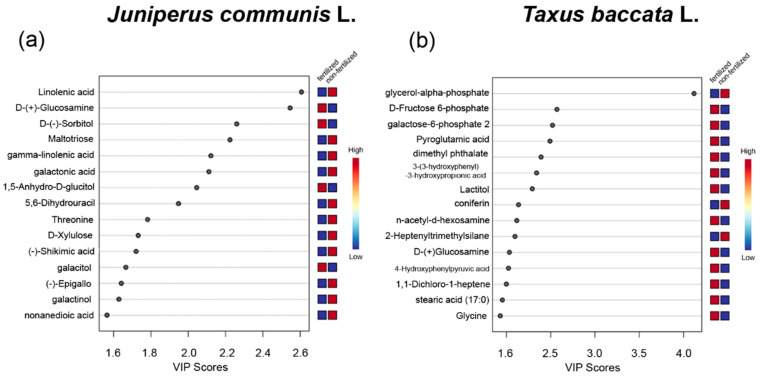
Results of the metabolome analysis of (**a**) *Juniperus communis* L. and (**b**) *Taxus baccata* L. pollen grains. VIP scores show the top 15 important metabolites that contribute the most to the PLS-DA plots of pollen grains. Colors in the variable importance in the projection plot represent relative intensities, where red and blue symbolize higher and lower values, respectively.

*J. communis* fertilizer-supplemented plants produced pollen grains with lower concentrations of fatty acids (linolenic acid and γ-linolenic acid), organic acids (galactonic acid, 3-hydroxyisovaleric acid, and shikimic acid), oligosaccharide (maltotriose), monosaccharide (D-xylulose and D-(−)-sorbitol), amino acid (threonine), and 5,6-dihydrouracil but higher concentrations of monosaccharide (D-(+)-glucosamine, 1,5-anhydro-D-glucitol), and pyrimidine (5-methylcytosine). Linolenic acid and D-(+)-glucosamine were indicated as the most important compounds identified by PLS-DA (Figure 3a).

In *T. baccata*, on the basis of unpaired fold change analysis with an FC threshold equal to 2.0, we also observed the presence of metabolites with at least two-fold differences between pollen grains of fertilized and non-fertilized plants. Pollen grains collected from *T. baccata* fertilized plants were characterized by the presence of 50 downregulated and 68 upregulated metabolites compared with their non-fertilized counterparts (Table S4). However, two-sample t-test analysis with equal group variance of raw data pointed to 21 metabolites that were differentially expressed between experimental groups (*p* < 0.05), and one of them (glycerol-α-phosphate) could be classified as differential when the data were analyzed based on FDR. Combining the results from fold change analysis and t-tests in a volcano plot allowed the selection of 6 downregulated and 15 upregulated metabolites when raw data were used (Table 1 and Table S1), and none when based on FDR (*p* < 0.05).

*Taxus baccata* fertilized plants produced pollen grains with lower concentrations of glycerol-α-phosphate, cysteamine, and sugar (allose), organic acids (quinic acid and L-ascorbic acid), and trisaccharide but higher concentrations of amino acids (isoleucine, glycine and 5-methyl-DL-alanine, and L(+)-cystathionine), organic acids (4-hydroxybenzoic acid, nicotinic acid, methylmalonic acid, 3-hydroxyisovaleric acid, and pyroglutamic acid), and derivatives such as n-acetyl-d-hexosamine, dimethyl phthalate, galactose-6-phosphate, and D-fructose-6-phosphate. Glycerol-α-phosphate was indicated as the most important compound identified by PLS-DA (Figure 3b).

**Figure 4:**
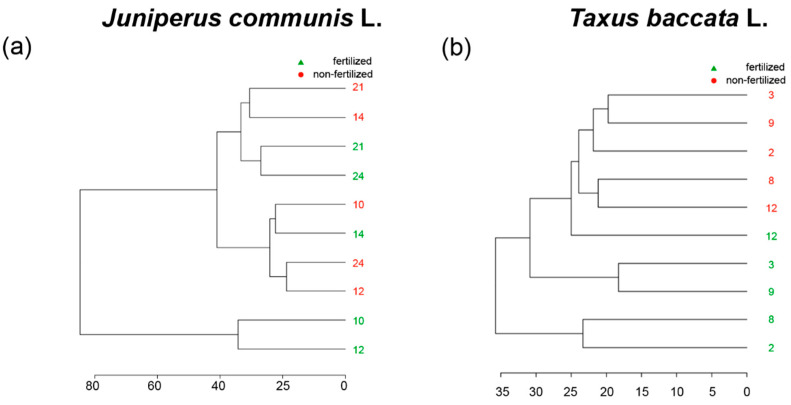
Dendrogram of the metabolome of (**a**) *Juniperus communis* L. and (**b**) *Taxus baccata* L. pollen grains collected from fertilized and non-fertilized male plants, generated with measured Euclidean distances and ward.D algorithm. Numbers in green indicate number of genotypes grown in fertilized conditions, whereas numbers in red indicate number of genotypes grown in non-fertilized conditions.

**Figure 5:**
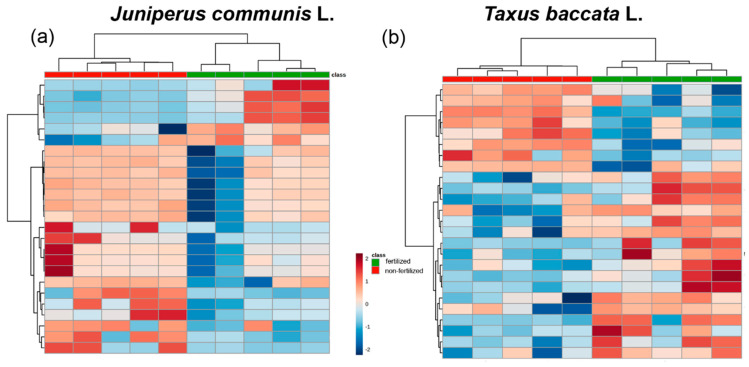
Results of (**a**) *Juniperus communis* L. and (**b**) *Taxus baccata* L. pollen grain metabolomes collected from analysis of long-term-fertilized (green) and non-fertilized (red) male plants with hierarchical clustering in the form of heat map prepared with measured Euclidean distance and ward.D clustering algorithm. The colored boxes indicate the relative concentration of the corresponding metabolite in each analyzed sample, where red and blue symbolize higher and lower values, respectively.

Although hierarchical clustering in the form of a dendrogram as well as heat map showed that both the genotype and the environment impacted the metabolites in pollen grains, there was no unique influence of genotype and/or environment in *J. communis* (Figure 4a and Figure 5a). Hierarchical clustering in the form of a dendrogram, as well as a heat map, showed that the environment had a significant impact on the profiles of metabolites in pollen grains of *T. baccata* (Figure 4b and Figure 5b).

## 3. Discussion

It has been shown that nutrient soil availability related to fertilizer supplementation impacts the in vitro germination abilities of pollen grains and, thus, pollen grains produced by long-term fertilizer-supplemented male plants of both species. In this study, we compared the metabolomic profiles of the mature pollen grains of two gymnosperm species, *T. baccata* and *J. communis*, collected from vegetatively propagated clone plants grown in long-term pot experiments within differentiated fertilizer conditions. We identified and quantified 224 metabolites in pollen grains and according to metabolome profiles, and some observed changes were related to the different fertilizer conditions. Moreover, differentiated metabolites can be a fingerprint of higher nitrogen content and a lower C:N ratio in fertilized pollen grains [11,12].

Pollen grains are male microgametophytes and play a crucial role in the sexual reproduction of plants. Pollen grain development is a complex process that depends on many factors, including environmental factors. In both studied species, the development of male reproductive structures starts in the late summer of the year before pollination. In the case of *T. baccata*, pollination starts in February/March, whereas *J. communis* pollen grains are released in April/May. During the period of pollen grain development, resources required for germination and pollen tube growth—such as sugars, lipids, and proteins—are accumulated [31]. Anemophilous pollen grains tend to accumulate starch as their main energy reserve, whereas entomophilous pollen grains tend to accumulate relatively more storage oils (after [32]). *J. communis* and *T. baccata* pollen grains are distributed by wind and, therefore, starch is considered as their the main energy reserve. In this study, pollen grains were collected from vegetatively propagated clone plants grown under long-term fertilizer supplementation in the same experimental conditions, such as regarding light, water, etc. Moreover, pollen grains produced by individuals that received fertilizer supplementation each year were previously characterized as having a significantly lower germination rate in vitro and higher nitrogen content. Although pollen grains from fertilized plants had a 0.2% increased ratio of nitrogen for *T. baccata* and a 0.41% increase for *J. communis* and similar carbon content when compared with the pollen grains of non-fertilized plants, the germination ability was decreased by 15% [10] for *T. baccata* and by 33% for *J. communis* [11]. However, this could be related to the low C:N ratio and the decrease in the enzymatic activity of enzymes involved in carbohydrate metabolism [33], as well as the strategy of resource allocation in pollen as the primary source of energy [34].

Information about the metabolomes of pollen grains is mostly related to the developmental potential of pollen grains [14,27]. Our study is an attempt to fill the gap in knowledge regarding the impact of environmental factors on the quality of pollen grains in relation to metabolite profiles and to understand the reasons for the lower germination ability of pollen grains produced by male plants supplemented with fertilizer. Therefore, our obtained results can be used as a fingerprint for pollen grains that develop in plants grown in stress conditions of higher nitrogen and phosphorus deposition in soil. Although we did not observe a similar pattern of changes in the pollen metabolome profiles of *J. communis* and *T. baccata* due to nutritional availability, this could be related to the different environmental requirements of species, as *T. baccata* prefers rich habitats, whereas *J. communis* is a pioneer species [29,35]. Previously published data also point to different patterns of change in the metabolite profiles within the seeds of these two species in response to different nutritional treatments. Seeds produced by long-term-fertilized plants compared with their non-fertilized counterparts are characterized by the presence of 18 differential metabolites in *J. communis* and none in *T. baccata* plants [10]. In both pollen grains and seeds, the differential metabolites were related to N detoxification and/or N storage.

**Figure 6:**
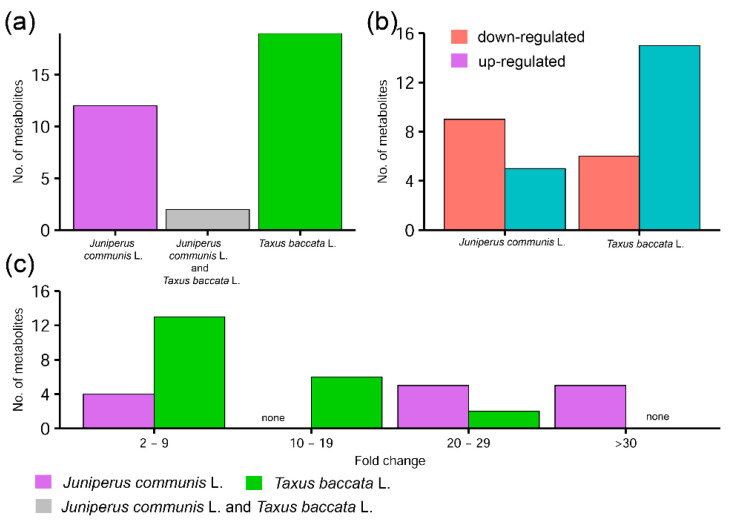
Comparison of number of (**a**) total differentiated metabolites of *J. communis* and *T. baccata* pollen grains; (**b**) number of up- and downregulated differentiated metabolites; (**c**) number of metabolites in fold-change classes.

We have not observed a similar pattern of fertilizer supplementation on the metabolome profiles of *J. communis* and *T. baccata* pollen grains considering both qualitative and quantitative aspects (Table 1). However, hierarchical clustering pointed to a significant impact of the environment on the *T. baccata* metabolome. All the samples of pollen grains produced by *T. baccata* plants clustered together, i.e., fertilized as well as non-fertilized. However, not all analyzed samples within the treatment group were characterized by the same quantitative changes and, thus, possible genotype impacts could also be observed (Figure 5a,b). However, it is also worth noting that *J. communis* plants grown in fertilized conditions had a more than 20- or even 30-fold increase or decrease in the relative content of some metabolites when clone individuals grown in two different nutritional regimes were compared. In *T. baccata*, the fold-change was up to a 20-fold difference (Figure 6). This could be partially due to fact that *J. communis* is a pioneer species that evolved to grow in environments with low quality of soils. Therefore, *J. communis* could be exposed to higher abiotic stress than *T. baccata*. Higher nutrition availability could cause osmotic stress in the pollen grains of both species; thus, the accumulation of soluble sugars was observed [36].

When only metabolites described as differentiated between treatment groups in each species were selected and compared, we observed that both species had different metabolite profiles (Table 1, Figure 6). However, two metabolites (5-methylcytosine and 3-hydroxyisovaleric acid) were present in both species. Firstly, 3-hydroxyisovaleric acid was downregulated in *J. communis* but upregulated in *T. baccata*. However, in both species, 5-methylcytosine was observed to be significantly increased in pollen grains collected from fertilizer-supplemented plants. We observed a 3.4-fold increase in *J. communis* and a 2.3-fold increase in *T. baccata* of 5-methylcytosine in the pollen grains of fertilized plants. Further, 5-methylcytosine is an epigenetic marker that plays a pivotal role in the transcriptional regulation of genes in plants and mammals, as well as the silencing of transposable elements. Studies based on *A. thaliana* have shown that male fertility and therefore sexual reproduction in plants is dependent on the active DNA demethylation of genes that control pollen tube function [37]. These increases could be related to the widespread methylation of numerous genes and therefore the significantly lower ratio of pollen grain germination or even lack of pollen tube formation during in vitro germination [11,12].

**Figure 7:**
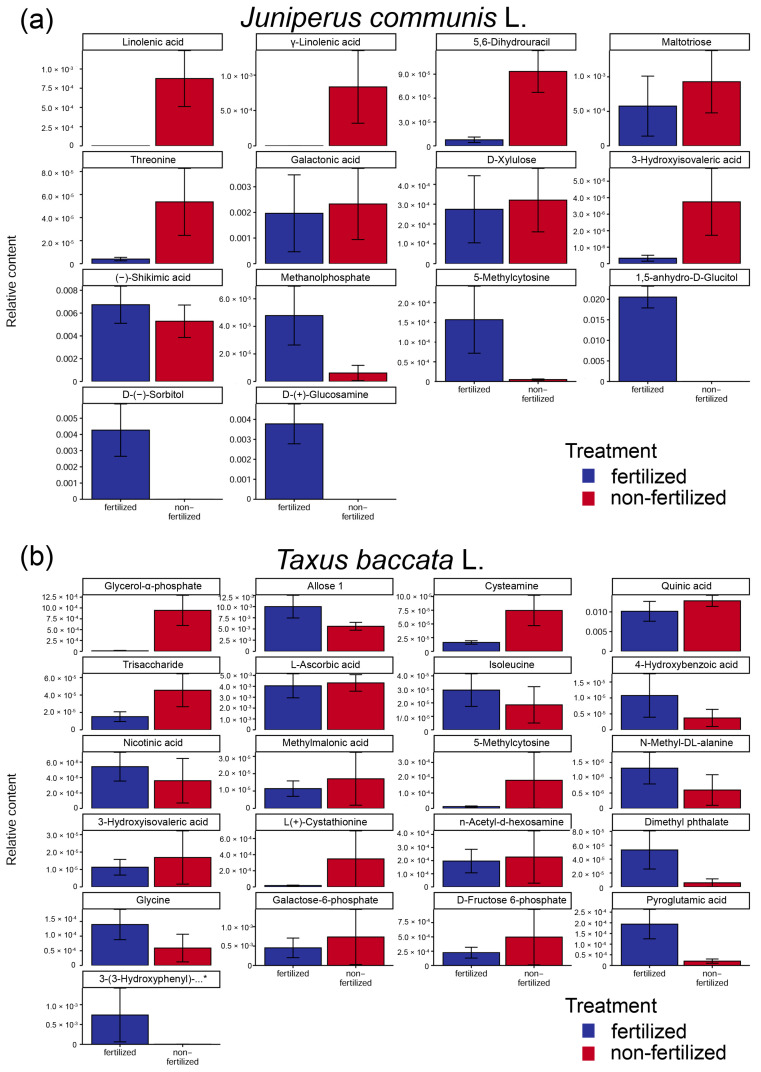
Y-axis shows the differences among the relative content of differentiated metabolites between pollen grains produced by long-term fertilizer-supplemented male individuals of (**a**) *Juniperus communis* L. and (**b**) *T. baccata* plants. Dark blue and red color shows the relative content of metabolites in pollen grains produced by fertilized and non-fertilized plants, respectively. 3-(3-Hydroxyphenyl)-…* indicates 3-(3-Hydroxyphenyl)-3-hydroxypropionic acid. Vertical bars represent standard error among five independent replicates. Data are the mean ± SEM of five replicates. All metabolites were significantly different at *p* < 0.05.

Fertilizer supplementation impacts carbohydrate metabolism in *J. communis* and *T. baccata* pollen grains (Figure 7). The main sources of pollen grains’ sugars present in *J. communis* pollen grains are galactose, glucose, arabinose, and xylose [38]. *J. communis* pollen grains from fertilized male individuals had a more than 20-fold decrease in maltotriose and D-xylulose, as well as the sugar acid galactonic acid, in addition to a more than 30-fold increase in sugar acids: D-(−)-sorbitol and 1,5-anhydro-D-glucitol. Sorbitol is a sugar alcohol whose primary photosynthetic products are involved in responses to stress, low-temperature, or salt stress [39]. The metabolite profiles of *T. baccata* pollen grains of fertilized plants were characterized by a more than two-fold decrease in allose, a two-fold increase in methylmalonic acid, a 13-fold increase in n-acetyl-d-hexosamine, and more than 15-fold increases in galactose-6-phosphate, D-fructose 6-phosphate, and pyroglutamic acid. Moreover, a more than 20-fold increase in 3-(3-hydroxyphenyl)-3-hydroxypropionic acid was also observed. Individuals of both species produced pollen grains with significantly high content of sugars and sugar acids, which could serve as energy resources during germination processes.

We also observed changes within fatty acid metabolism, with more than 35-fold decreases in linolenic acid and γ-linolenic acid in *J. communis* (Table 1, Figure 7). Moreover, we observed a 20-fold decrease in glycerol-α-phosphate, which is a starting material for the de novo synthesis of lipids that could also act as antioxidants in *T. baccata* pollen grains of fertilizer-supplemented plants. Lipidic or lipid-derived structures are an important component of all pollen grains, e.g., extracellular pollen walls, intracellular membrane systems, and storage oil bodies [32]. The lower content of linolenic acids and gamma-linolenic acid observed in *J*. *communis* is in line with the results obtained in *Arabidopsis thaliana* showing that linolenic acid is a critical requirement for pollen development. *A. thaliana* mutant plants lacking the production of linolenic acid produce pollen grains that are able to mature to the tricellular stage but experience significantly decreased pollen grain development beyond this stage, as well as seed production [40]. Similarly, higher levels of linolenic acid are associated with pollen tube formation [41]. Therefore, the low content of unsaturated fatty acids in pollen grains from fertilized *J. communis* plants could be one of the causes of the low germination ability of pollen grains under in vitro conditions. Moreover, it could also result from a higher level of free radicals within the tissue of fertilized plants, caused by the higher nitrogen content in needles [42], as well as in dry, mature pollen grains [11], compared with their non-fertilized counterparts. Higher nitrogen content and a lower C:N ratio in pollen grains can affect the maintenance of homeostasis by increasing the level of reactive nitrogen species (RNS) [43]. Although RNS, similarly to reactive oxygen species (ROS), are involved in maintaining homeostasis at the cellular level, they are also involved in various biological reactions, such as nitration, nitrosation, oxidation, and nitrosylation.

The metabolism of amino acids and amino acid derivatives in *J. communis* and *T. baccata* pollen grains was also affected by fertilizer supplementation. *J. communis* pollen grains from fertilized plants were characterized by a 35-fold increase in glucosamine and more than 20-fold decreases in 3-hydroxyisovaleric acid and threonine (Table 1, Figure 7). Glucosamine, an amino sugar, is a prominent precursor of all nitrogen-containing sugars and is involved in the biochemical synthesis of glycosylated proteins and lipids. Threonine and its metabolites have a significant role in chemical defense against abiotic stress, such as from salt and drought, and its metabolites are also involved in cell division and plant growth and development [44]. *T. baccata* pollen grains from fertilizer-supplemented individuals had decreased cysteamine content and increased content of N-methyl-DL-alanine (>two-fold), 3-hydrohyisovaleric acid, L(+)-cystathionine (>3-fold), glycine (>14-fold), and pyroglutamic acid (>16-fold). The higher pollen grain nitrogen content in both species [12] could be related to the higher ratio of metabolites containing nitrogen in their structure, e.g., pyroglutamic acid, n-acetyl-α-hexosamine, and glycine. Pyroglutamic acid, which is also known as 5-oxoproline, is a ubiquitous amino acid involved in abiotic stress responses.

Moreover, pollen grains of fertilized *T. baccata* could be linked with environmental pollutants; thus, samples had a 10-fold increase in diethyl phthalate (DEP). DEP is considered to have a toxic effect on human health [45] but, however, has no effect on pollen grain germination and tube formation [46].

Long-term fertilizer supplementation had a significant impact on pollen grains’ energy metabolism as some of the carbohydrates were affected, as well as pollen grains’ development and resistance against stresses as the content of some of amino acids and lipids was also changed. Therefore, according to the obtained results, it can be pointed out that the increased deposition of nitrogen, phosphorus, or sulfur in the soil environment, and their higher accumulation in seeds and/or pollen grains, can result in decreases in plant reproductive features. A higher level of nitrogen in the pollen grains of both species is related to a decrease in the germination ability of pollen grains and can be marked by the higher content of metabolites involved in the metabolism of glutathione, amino acids

(glycine, serine, and threonine; valine, leucine, and isoleucine; and cysteine and methionine), and sugars.

## 4. Materials and Methods

The study was conducted on male individuals of *Taxus baccata* L. and *Juniperus communis* L. plants that were obtained by rooting, as previously described [11,12]. Plants were grown in two different nutritional conditions from 2013 to 2018. The experiment was conducted on plants grown in two levels of nutrient conditions, since half of the plants received an application of fertilizer each year in March. Osmocote Exact 5-6 M (ICL, Tel Aviv, Israel) fertilizer was applied at the recommended dose of 6 g/L, and the fertilized plants received 0.75 g N (0.33 g N-NO_3_, 0.42 g N-NH_4_), 0.45 g P_2_O_5_, 0.6 g K_2_O, 0.125 g MgO, and microelements (22.5 mg Fe, 3.0 mg Mn, 1.0 mg B, 2.5 mg Cu, 1.0 mg Mo, and 0.75 mg Zn) per liter of soil. The control group of non-fertilized plants was grown without the use of any fertilizer or other supplements. Plants were grown in identical light, soil, and water conditions. Plants produced pollen grains that were characterized by different in vitro germination abilities, as was previously shown, and non-fertilized plants produced higher-quality pollen grains compared with their fertilized counterparts [11,12].

**Figure 8:**
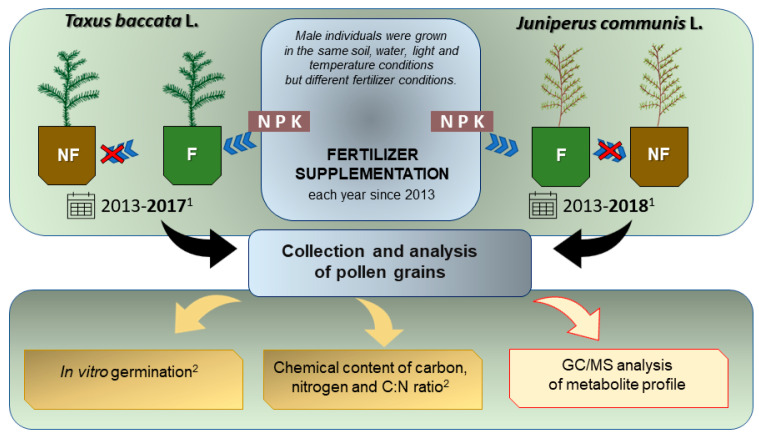
Experimental design. 1—year of pollen grains collection; 2—data were presented in Pers-Kamczyc et al. [11,12].

Materials used for metabolomic analysis were collected from *Taxus baccata* plants in March 2016 and from *Juniperus communis* plants in May 2017, at the same time as the pollen grains that were used in our previous studies [11,12]. A biological replicate consisted of a pool of pollen grains derived from male flowers of three to five plants of the same genotype, and, each time, samples were collected from plants of the same genotype grown under two different experimental conditions. We analyzed five biological replicates for each of the treatment conditions. Pollen grains were weighed and frozen in liquid nitrogen and stored at −80 °C until further analysis (Figure 8).

We used 150 mg of pollen grains per biological sample for metabolome analysis, and recently published extraction protocols [17,47] were followed for preparing samples for GC–MS analysis. Briefly, the pollen grain sample (150 mg) was weighed and ground into a fine powder in liquid nitrogen. Next, 1 mL ice-cold methanol/chloroform solution (3:1, *v*/*v*) was added. Samples were then vortexed at 6000 rpm for 15 s (repeated three times). After this, samples were centrifuged at 15,000× *g* for 10 min, and 300 µL supernatant of each sample was transferred to a new vial and vacuum-dried at room temperature.

For derivatization, 80 µL of 20 mg/mL methoxyamine hydrochloride (dissolved in pyridine) was added to the vial, and samples were then transferred into an oven and incubated at 37 °C for 1.5 h. Then, 80 µL of MSTFA (N-methyl-N-(trimethylsilyl)trifluoroacetamide) was added, followed by incubation at 37 °C for 1 h. Supernatants of all samples were mixed, vacuum-dried, and derivatized in the same manner to prepare QC samples. Derived extract (1 µL) of each sample was analyzed using a TRACE 1310 GC oven with TSQ8000 triplequad MS from Thermo Scientific (Waltham, Massachusetts, USA), coupled with a DB-5MS capillary column (30 m × 0.25 mm × 0.25 µm, J&W Scientific). Chromatographic separation conditions in gradient mode were kept as follows: 70 °C for 2 min, followed by 10 °C/min up to 300 °C, at 300 °C (10 min). A PTV injector was used for sample injection with a temperature gradient from 40 to 250 °C, the column interface temperature was maintained at 250 °C, and the source temperature was 250 °C. The ion source was operating in the m/z range 50–850 in EI positive mode and with electron energy set to 70 eV and a carrier gas (He) flow rate of 1.2 mL/min. The mixture of Supelco C7–C40 saturated alkane standard was run before the samples to calculate the retention index of each feature. The QC samples were run at the start, middle, and end of the analysis (every 5 samples).

Raw MS data were converted to abf format and analyzed using MSDial software package v. 3.96. To eliminate the retention time (Rt) shift and to determine the retention indexes (RI) for each compound, the alkane series mixture (C-10 to C-36) was injected into the GC–MS system. Identified artifacts (alkanes, column bleed, plasticizers, MSTFA, and reagents) were excluded from further analyses. Obtained normalized (using total ion current (TIC) approach and LOWESS algorithm) results were then exported to Excel for pre-formatting and then subjected to statistical analyses. Analysis was conducted by the Laboratory of Mass Spectrometry, Institute of Bioorganic Chemistry, Polish Academy of Sciences.

Then, a data matrix containing the feature name (identified compound name), sample information (four biological replicates per sample), and relative abundance (calculated by peak area) was prepared and submitted to MetaboAnalyst (https://www.metaboanalyst.ca/, access on 5 September 2022). All data collected for *T. baccata* and *J. communis* plants were separately analyzed for each species. Data obtained from GC–MS describing pollen grain metabolites, principal component analysis, and correlation and pattern analyses were assessed using MetaboAnalyst 5.0, a comprehensive tool suite for metabolomic data analysis (http://metaboanalyst.ca/, access on 5 September 2022; [48]), following data log_10_ transformation and scaling manipulations. For metabolomic analysis, five biological replicates per treatment were used. Each time, pollen grains were sampled from the same male genotype that were grown in different treatment conditions.

The data matrix was prepared for three categories of normalization, including normalization by median, square root data transformation, and Pareto via online data analysis software MetaboAnalyst and using the embedded algorithm. The differential metabolites were screened by parameters including fold change (FC > |2.0|), the variable influence on projection (VIP, VIP value > 1), and *p*-value < 0.05. The VIP value > 1 for a metabolite demonstrates that it contributes greatly to the separation of sample groups in the PLS-DA models. Then, these ‘candidates’ were marked for pathway identification to explore their biological roles during seed germination.

Metabolic pathway enrichment analysis was used to interpret the biological significance associated with metabolites detected in pollen grains. Pathway analysis was conducted on the basis of the Kyoto Encyclopedia of Genes and Genomes (KEGG) metabolic pathway analysis with MetaboAnalyst 5.0. The enrichment analysis considered the metabolomic pathway ID number, pathway name, the number of metabolites involved in the pathway, the *p*-value of the metabolic pathway, the false discovery rate (FDR), the corrected *p*-value, and -log (*p*-value).

## 5. Conclusions

In summary, male reproductive performance, considered on the basis of pollen grain quality, was disrupted by the long-term deposition of nitrogen, phosphorus, and other minerals in two gymnosperm species, *Juniperus communis* L. and *Taxus baccata* L. Pollen grain carbohydrate, amino acid, and fatty acid metabolism were significantly affected by the high availability of N and P. This study also showed that the impact of the long-term fertilization of long-living trees can have a significant effect on pollen grain compounds and seed production. Nevertheless, in the face of the increasing deposition of nitrogen, phosphorus, and sulfur in soil due to increasing fertilizer usage, further studies performed on a wide range of species are needed to understand the specific relationship between the soil nutrient availability and pollen development and germination ability in each of these species.

## Figures and Tables

**Table 1:** Differential metabolites of *Juniperus communis* L. and *Taxus baccata* L. pollen grains produced by comparison between plants grown in fertilized (F) and non-fertilized (NF) conditions.

	Compound	Log2 (FC F/NF) ^1^	*p*-Value	VIP Value ^2^
*Juniperus communis* L.	Linolenic acid	−37.13	0.015	2.157
	γ-Linolenic acid	−36.54	0.036	1.753
	5,6-Dihydrouracil	−27.03	0.033	1.594
	Maltotriose	−26.86	0.008	2.181
	Threonine	−26.71	0.044	1.429
	Galactonic acid	−24.94	0.011	2.008
	D-Xylulose	−23.84	0.034	1.552
	3-Hydroxyisovaleric acid	−5.10	0.047	1.184
	(−)-Shikimic acid	−1.54	0.042	1.414
	Methanolphosphate	2.19	0.020	0.771
	5-Methylcytosine	3.39	0.019	0.818
	1,5-anhydro-D-Glucitol	30.12	0.018	2.480
	D-(−)-Sorbitol	31.54	0.006	2.607
	D-(+)-Glucosamine	34.64	0.002	2.826
*Taxus baccata* L.	Glycerol-α-phosphate	−21.81	1.93 × 10^−6^	3.927
	Allose 1	−2.66	0.006	1.367
	Cysteamine	−2.39	0.002	1.284
	Quinic acid	−2.06	0.004	1.036
	Trisaccharide	−2.00	0.008	0.991
	L-Ascorbic acid	−1.93	0.009	1.087
	Isoleucine	1.19	0.039	0.706
	4-Hydroxybenzoic acid	1.25	0.018	0.799
	Nicotinic acid	2.04	0.022	1.194
	Methylmalonic acid	2.25	0.029	0.874
	5-Methylcytosine	2.26	0.036	0.875
	N-Methyl-DL-alanine	2.50	0.013	1.250
	3-Hydroxyisovaleric acid	2.50	0.016	1.106
	L(+)-Cystathionine	3.90	0.027	1.157
	n-Acetyl-d-hexosamine	13.71	0.026	1.995
	Dimethyl phthalate	13.85	0.026	2.253
	Glycine	14.65	0.047	1.855
	Galactose-6-phosphate	15.57	0.016	2.370
	D-Fructose 6-phosphate	16.09	0.015	2.422
	Pyroglutamic acid	16.70	0.014	2.352
	3-(3-Hydroxyphenyl)- 3-hydroxypropionic acid	20.38	0.037	2.246

^1^ Fold changes in different metabolites between F and NF pollen grains are reported as Log2 (FC F/NF). ^2^ VIP value—weighted sum of squares of the PLS weight, which indicates the importance of the variable to the whole model.

## Data Availability

Not applicable.

## Supplementary Materials

The following supporting information can be downloaded at https://www.mdpi.com/article/10.3390/ijms232214105/s1.
